# Editorial: Cardiac optogenetics: Using light to observe and excite the heart

**DOI:** 10.3389/fphys.2022.1031062

**Published:** 2022-10-11

**Authors:** Tobias Bruegmann, Godfrey L. Smith, Stephan E. Lehnart

**Affiliations:** ^1^ Institute for Cardiovascular Physiology, University Medical Center Goettingen, Goettingen, Germany; ^2^ Cluster of Excellence “Multiscale Bioimaging: From Molecular Machines to Networks of Excitable Cells“ (MBExC), University of Goettingen, Goettingen, Germany; ^3^ DZHK (German Centre for Cardiovascular Research), Partner Site Goettingen, Goettingen, Germany; ^4^ School of Cardiovascular and Metabolic Health, University of Glasgow, Glasgow, United Kingdom; ^5^ Department of Cardiology and Pulmonology, Heart Research Center Göttingen, University Medical Center Göttingen, Georg August University of Göttingen, Göttingen, Germany; ^6^ Collaborative Research Center SFB1190 “Compartmental Gates and Contact Sites in Cells“, University of Goettingen, Goettingen, Germany

**Keywords:** optogenetics, channelrhodopsins, cardiac arrhythmia, imaging, heart

This is the editorial to the special edition “Cardiac optogenetics: using light to observe and excite the heart.

The application of fluorescent voltage sensitive dyes to study excitable cells was established 50 years ago (Tasaki et al., 1969) but only recently has chemical and imaging technology developed sufficiently for its mainstream use. In contrast, the field of cardiac optogenetics was established only a decade ago by publications demonstrating light-mediated excitation of the heart in mice (Bruegmann et al., 2010), zebrafish (Arrenberg et al., 2010) and cardiomyocytes *in vitro* (Abilez et al., 2011; Jia et al., 2011). Ever since, the subject of optogenetics has expanded to encompass a number of different applications. Early translational approaches considered light-mediated cardiac resynchronization therapy (Nussinovitch and Gepstein 2015), defibrillation (Bruegmann et al., 2016; Crocini et al., 2016; Nyns et al., 2017) and cardioversion (Bruegmann et al., 2018; Nyns et al., 2019). In this regard, Diaz-Maue et al. developed a mesh of electrodes and LEDs to correlate electrical rotor activity during arrhythmias with defibrillation efficacy of optogenetic stimulation and Patrick Boyle’s group explored in simulations the use and application of anion conducting channelrhodopsins (Ochs et al.). While leading also to depolarization in cardiomyocytes (Kopton et al., 2018), the much larger ion conductance improved the efficiency of optogenetic defibrillation, which is an intriguing result directly demanding experimental verification. State-of-the-art solutions for one of the biggest hurdles of translation, the development of implantable light devices has been expertly summarized and thoughtfully discussed by Igor Efimov’s group in this issue (Madrid et al.).

One big advantage of optogenetic stimulation is the cell type-specific expression providing not only the chance for pain-free stimulation but also to characterize the specific role of different cell types by cell type-specific (e.g. ventricular cardiomyocytes versus Purkinje fibers) stimulation (Zaglia et al., 2015; Hulsmans et al., 2017; Wang et al., 2017) as well as imaging (Quinn et al., 2016) within the heart. In this context, Zaglia and Mongilo have expertly summarized new developments of optogenetic stimulation to assess the function and role of the intracardiac nervous system (Scalco et al.), emphasizing the heterocellular, increasingly complex composition and functions of specific cardiac tissues, and further raising the importance of optogenetic strategies to explore these.

Moreover, the range of applications of voltage-sensitive dyes combined with optogenetic stimulation in basic cardiovascular research have been critically reviewed by a group of scientists from the European Society of Cardiology Working Group for Cardiac Cellular Electrophysiology (Mullenbroich et al.). The review examines many of the novel techniques that optical physics have provided to extend the use of optical probes and actuators while also posing the next set of challenges to be addressed to extend further the applicability of these techniques. In this content, Jan Lebert and Jan Christoph present new algorithms for the analysis of voltage imaging with motion tracking stabilization to avoid the alterations of cardiac electrophysiology by contraction inhibitors with significant side effects (Lebert et al., 2022). Furthermore, Wegener and colleagues took advantage of transgenic biosensor mouse models to analyze the cytosolic and mitochondrial glutathione redox potential in single cardiomyocytes and the intact heart. Thereby they were able to show that Ca^2+^ leak caused by a ryanodine receptor missense mutation increases mitochondrial energy demand and ROS production under conditions of catecholaminergic stress (Wegener et al.). Finally, Philipp Sasse’s group expanded the optogenetic toolbox for cardiac research demonstrating that the human coneopsin allows to control G_i_ signaling in embryonic stem cell derived cardiomyocytes (Cokic et al.). Thus, the three canonical G-protein pathways of the heart (Makowka et al., 2019; Wagdi et al., 2022) can now be investigated and their underlying kinetics precisely determined.

Daniel Pijnappel’s group characterized potential long term effects of optogenetic stimulation via channelrhodopsins (Ordog et al.) and the groups of Christina Schüler and Leonardo Sacconi developed new methods and platforms for cardiac toxicity screening (Credi et al.; Engel et al.) which is one of the evolving cardiac research fields in which the use of optogenetic stimulation is becoming more and more standard (Klimas et al., 2016; Lapp et al., 2017; Rehnelt et al., 2017). Notably, the optical transparency of zebrafish and their rather easy handling as animal model as well as fast generation and genetic manipulation of transgenic animals, has led to their increasing use to study heart function using optogenetics (Baillie et al.), whereas intact hearts from mice and even bigger animals have to be cleared for imaging of the cell composition and structure (Olianti et al., 2022; Ren et al.).

In conclusion, this special issue is covering the broad range of dye-based imaging and optogenetic applications in the heart and the advances made in each branch of the subject by new technical improvements and comprehensive reviews. We hope that we and all contributors are able to trigger further interest in and advance the use of optogenetic stimulation and imaging within the field of cardiac research.
